# Supplementary material for: Impact of Adjuvant Atezolizumab on Recurrences Avoided and Treatment Cost Savings for Patients with Stage II-IIIA Non-Small Cell Lung Cancer in Canada
Source: Curr Oncol. 2024 Jun 7;31(6):3301–10. doi: 10.3390/curroncol31060251 (PMC11202522; doi:10.3390/curroncol31060251)
Supplement: Supplementary file 1 [file curroncol-31-00251-s001.zip › curroncol-2997400-supplementary.pdf]

Impact of adjuvant atezolizumab on recurrences avoided and treatment cost savings for patients with stage II-IIIA non-small cell lung cancer in Canada

SUPPLEMENTARY MATERIALS

Table S1. Clinical pathway inputs

Health state	Event	Input value	Source
DFS event	Locoregional recurrence	41.2%	IMpower010[1]
	Metastatic recurrence	51.6%	IMpower010[1]
	Death	7.2%	IMpower010[1]
Locoregional recurrence	Proceed with chemoradiotherapy +/- durvalumab treatment	80.0%	Assumption, clinical expert consultation
	If PFS event: disease progression	81.0%	NICE TA578[2]
Metastatic recurrence (1L)	Proceed with treatment	77.0%	Assumption, clinical expert consultation
	Receive pembrolizumab (eligible for IO therapy)	48.0%	Assumption, clinical expert consultation
	Receive cisplatin + vinorelbine (eligible for IO therapy)	0	Assumption, clinical expert consultation
	Receive pembrolizumab + cisplatin + pemetrexed (eligible for IO therapy)	52.0%	Assumption, clinical expert consultation
	Receive pembrolizumab (no IO therapy)	0	Assumption, clinical expert consultation
	Receive cisplatin + vinorelbine (no IO therapy)	100.0%	Assumption, clinical expert consultation
	Receive pembrolizumab + cisplatin + pemetrexed (no IO therapy)	0	Assumption, clinical expert consultation
	If PFS event: disease progression	82.2%	NICE TA584[3]
Metastatic recurrence (2L)	Proceed with docetaxel treatment	50.0%	Assumption, clinical expert consultation

1L, first line; 2L, second line; DFS, disease-free survival; IO, immuno-oncology; NICE, National Institute for Health and Care Excellence; PFS, progression-free survival; TA, technology appraisal.

Table S2. Locoregional and metastatic recurrence treatment costs

Treatment	Input	Value (2023 CAD)	Sources
Chemotherapy	Administration	$75.00	Ministry of Health (2023)[4]
Immunotherapy	Administration	$105.15	Ministry of Health (2023)[4]
Locoregional recurrence			
Radiotherapy	Dose (Gy)	66	
	Cost per fraction (2 Gy)	$255.20	Ministry of Health (2023)[4]
Chemotherapy	Drug 1<br>Dose<br>Cycles<br>Cost per mg	Cisplatin<br>80 mg/m^2<br>4<br>$2.70	Clinical consultation<br>CADTH (2021)[5]
	Drug 2<br>Dose<br>Cycles<br>Cost per mg	Vinorelbine<br>60 mg/m^2<br>4<br>$3.77	Clinical consultation<br>IQVIA DeltaPA Database[6]
	Drug 3<br>Dose<br>Cycles<br>Cost per mg	Durvalumab<br>10 mg/kg<br>20<br>$7.82	Antonia (2017)[7]<br>CADTH (2021)[5]
Metastatic recurrence (1L)			
Treatment option 1	Drug 1<br>Dose<br>Cycles<br>Cost per mg	Pembrolizumab<br>200 mg fixed<br>10<br>$44.00	Clinical consultation<br>Gandhi (2018)[8]<br>pCODR (2020)[9]
Treatment option 2	Drug 1<br>Dose<br>Cycles<br>Cost per mg	Cisplatin<br>80 mg/m^2<br>5<br>$2.70	Clinical consultation<br>Scagliotti (2008)[10]<br>Ministry of Health (2023)[4]
	Drug 2<br>Dose<br>Cycles<br>Cost per mg	Vinorelbine<br>60 mg/m^2<br>5<br>$3.77	Clinical consultation<br>Scagliotti (2008)[10]<br>IQVIA DeltaPA Database[6]
Treatment option 3	Drug 1<br>Dose<br>Cycles<br>Cost per mg	Pembrolizumab<br>200 mg fixed<br>10<br>$44.00	Clinical consultation<br>Gandhi (2018)[8]<br>pCODR (2020)[9]
	Drug 2<br>Dose<br>Cycles<br>Cost per mg	Pemetrexed<br>500 mg/m^2<br>9<br>$0.83	Clinical consultation<br>Gandhi (2018)[8]<br>pCODR (2020)[9]
	Drug 3<br>Dose<br>Cycles<br>Cost per mg	Cisplatin<br>80 mg/m^2<br>9<br>$2.70	Ministry of Health (2023)[4]
Metastatic recurrence (2L)			
	Drug 1<br>Dose<br>Cycles<br>Cost per mg	Docetaxel<br>75 mg/m^2<br>4<br>$11.42	Clinical consultation<br>Reck (2014)[11]<br>pCODR (2016)[12]

1L, first line; 2L, second line; CADTH, Canadian Agency for Drugs and Technologies in Health; pCODR, pan-Canadian Oncology Drug Review.

Table S3. Estimated events in the base case analysis (10 years, 2024-2034)

	Alberta	British Columbia	Manitoba	New Brunswick	Newfoundland and Labrador	Nova Scotia	Ontario	Prince Edward Island	Quebec	Saskatchewan	Yukon, Northwest Territories, Nunavut	Canada
Locoregional recurrences												
Atezolizumab	49	73	17	15	21	16	205	3	167	15	1	**583**
BSC	58	86	20	18	25	19	242	4	196	17	1	**686**
Recurrences avoided	9	13	3	3	4	3	36	1	30	3	0	**103**
Metastatic recurrences												
Atezolizumab	62	92	22	19	26	20	250	4	209	18	1	**723**
BSC	73	108	25	22	31	23	303	5	246	22	2	**859**
Recurrences avoided	11	16	4	3	5	3	52	1	37	3	0	**136**
Any recurrence												
Atezolizumab	111	165	39	34	47	36	456	7	376	33	2	**1306**
BSC	131	194	46	40	56	42	544	8	442	39	3	**1546**
Recurrences avoided	20	29	7	6	9	6	89	1	66	6	0	**240**
Deaths												
Atezolizumab	9	13	3	3	4	3	35	1	29	3	0	**101**
BSC	10	15	4	3	4	3	42	1	34	3	0	**120**

	Alberta	British Columbia	Manitoba	New Brunswick	Newfoundland and Labrador	Nova Scotia	Ontario	Prince Edward Island	Quebec	Saskatchewan	Yukon, Northwest Territories, Nunavut	Canada
Recurrences avoided	2	2	1	0	1	0	7	0	5	0	0	**19**
Any event												
Atezolizumab	120	178	42	37	51	39	491	7	405	36	3	**1407**
BSC	142	209	49	44	61	45	586	9	477	42	3	**1666**
Recurrences avoided	22	32	7	7	9	7	96	1	72	6	0	**259**

BSC, best supportive care.

Table S4. Estimated costs ($MM) of treating recurrences in the base case analysis (10 years, 2024-2034)

	**Alberta**	**British Columbia**	**Manitoba**	**New Brunswick**	**Newfoundland and Labrador**	**Nova Scotia**	**Ontario**	**Prince Edward Island**	**Quebec**	**Saskatchewan**	**Yukon, Northwest Territories, Nunavut**	**Canada**
Atezolizumab	$11.5	$17.0	$4.0	$3.6	$4.9	$3.7	$47.8	$0.7	$38.9	$3.4	$0.2	**$135.8**
BSC	$14.4	$21.2	$5.0	$4.4	$6.1	$4.6	$59.5	$0.9	$48.3	$4.2	$0.3	**$169.0**
Cost savings, atezolizumab vs BSC	$2.9	$4.2	$1.0	$0.9	$1.2	$0.9	$11.7	$0.2	$9.5	$0.8	$0.1	**$33.2**

BSC, best supportive care.

Figure S1. Calculation of the target population

$$N_t = \sum_{i=stage\ IIa}^{stage\ IIIa} I_t \cdot V_t \cdot S_{ti} \cdot P_t \cdot T_t \cdot B_t$$

Notation	Definition
N_t	Total number of eligible patients to include in TIM at time *t*
I_t	Total number of patients diagnosed with lung cancer at time *t*
V_t	Proportion of lung cancer patients at time *t* who have NSCLC
S_{ti}	Proportion of resected NSCLC patients at time *t* with stage *i* disease
P_t	Proportion of resected NSCLC patients at time *t* who are PD-L1+ (≥ 50%)
B_t	Proportion of resected NSCLC patients at time *t* who are ALK- and EGFR-
T_t	Proportion of resected NSCLC patients at time *t* receiving adjuvant treatment

NSCLC, non-small cell lung cancer; PD-L1, programmed cell death 1 ligand 1; TIM, treatment impact model.

Figure S2. Probability of a patient experiencing recurrence or dying

$$PA_x = 1 - \frac{DFSA_t}{DFSA_{t-1}}, PBs_x = 1 - \frac{DFSBs_t}{DFSBs_{t-1}}$$

Notation	Definition
PA_x	Probability of experiencing recurrence or death *x years* after adjuvant atezolizumab initiation
PBs_x	Probability of experiencing recurrence or death *x* years after best supportive care initiation
$DFSA_t$	Proportion of patients who are in DFS at time *t* (adjuvant atezolizumab arm IMpower010)
$DFSBs_t$	Proportion of patients who are in DFS at time *t* (best supportive care arm IMpower010)

DFS, disease-free survival.

Figure S3. Atezolizumab disease-free survival extrapolations

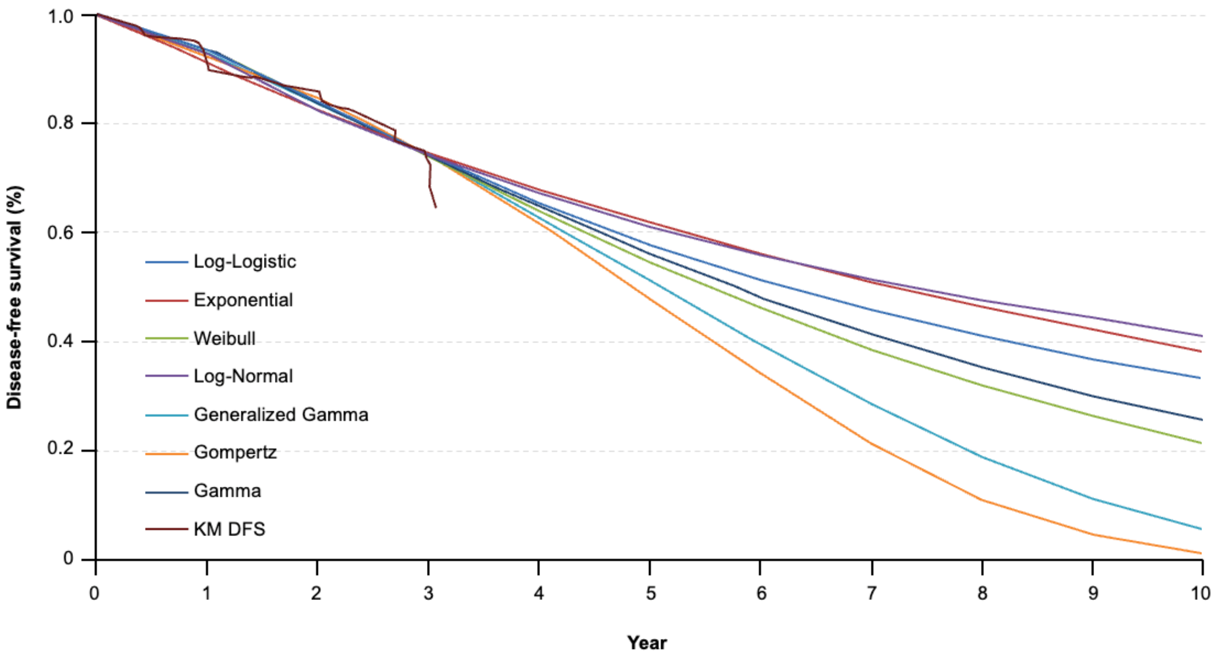

Figure S4. Best supportive care disease-free survival extrapolations

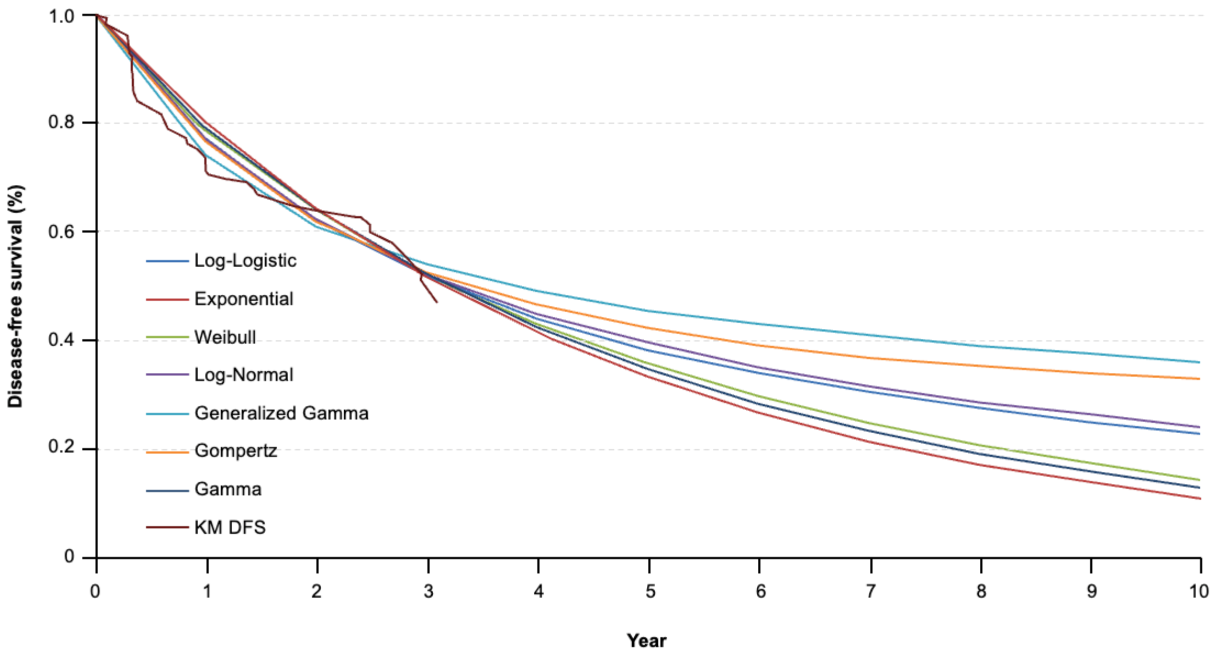

Figure S5. Number of patients experiencing recurrence or dying

$$TRA_t = \sum_{x=1}^{10} TA_{t,x} \quad where \quad TA_{t,x} = \begin{cases} x = 1 & ATZ_{t-1} \cdot PA_x \cdot R \\ x > 1 & \sum_{x=2}^{10} (ATZ_{t-x} - \sum_{y=1}^{9} RA_{t-y,x-y}) \cdot PA_x \cdot R \end{cases}$$

Notation	Definition
TRA_t	Number of patients on adjuvant atezolizumab who experience recurrence at time *t*
$TA_{t,x}$	Number of patients on adjuvant atezolizumab who experience recurrence at time *t* conditional on the number of years *x* they have been disease-free
$RA_{t,x}$	Number of patients on adjuvant atezolizumab who have experienced recurrence by time *t* conditional on experiencing it at time *t* and *x* years after initiation of adjuvant atezolizumab
R	Proportion of patients who had death as their DFS event (pooled across arms IMpower010)

BSC, best supportive care; DFS, disease-free survival.

Identical equations were used for the BSC treatment arm.

Figure S6. Estimated number of avoided recurrences with adjuvant atezolizumab compared with BSC by province, recurrence type, and uptake scenario (10 years, 2024-2034)

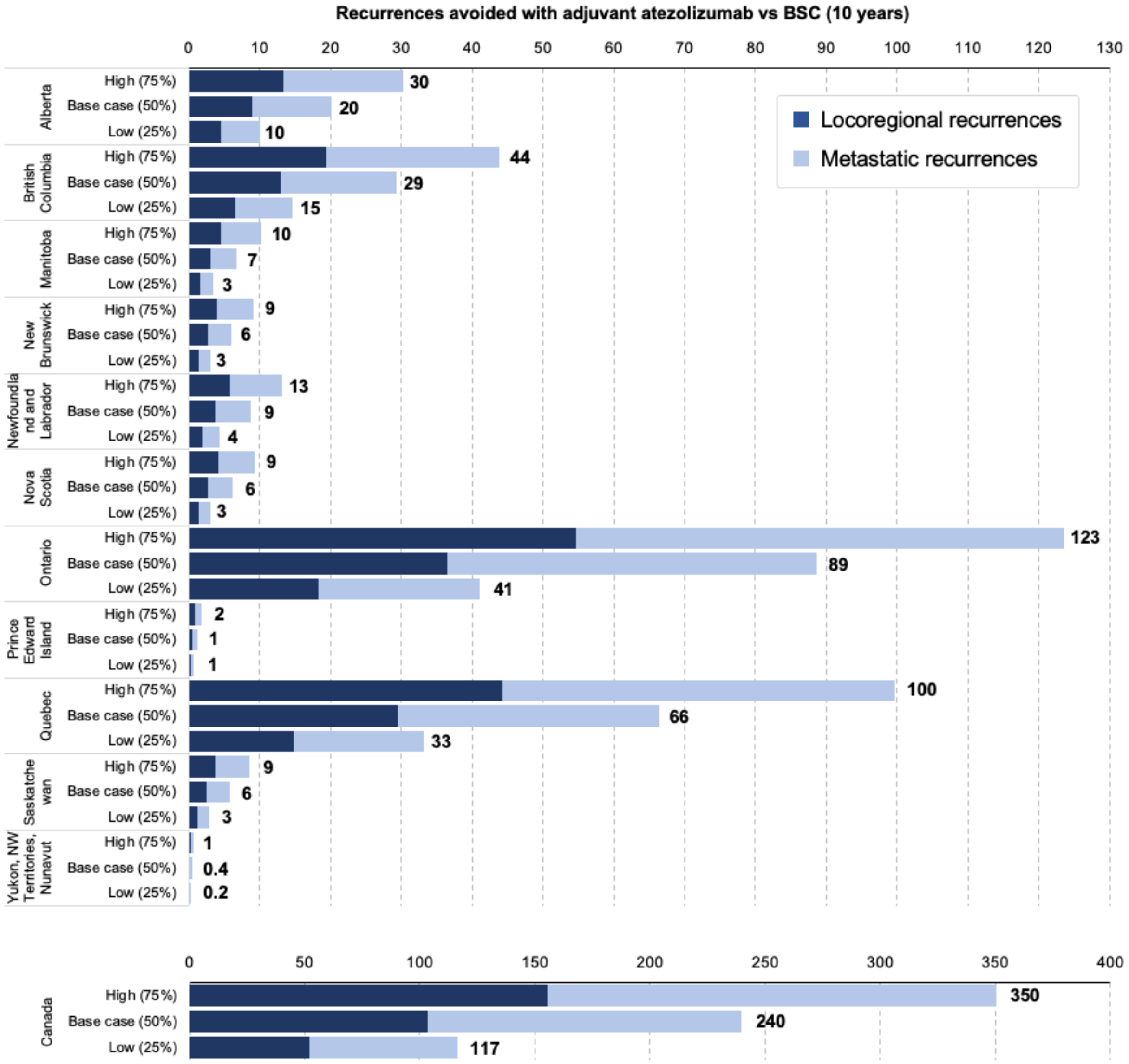

BSC, best supportive care.

Figure S7. Estimated differences in costs of treated recurrences with adjuvant atezolizumab compared with BSC by province and uptake scenario (10 years, 2024-2034)

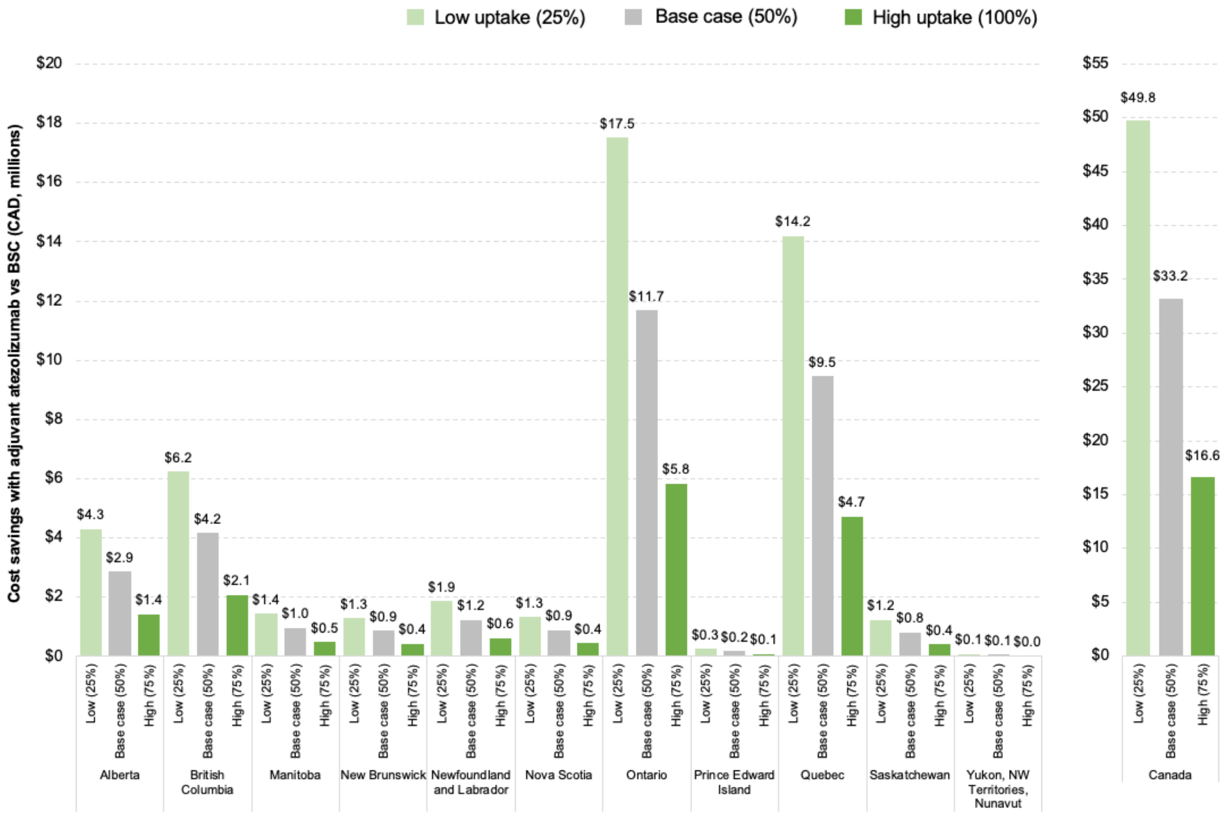

BSC, best supportive care.

Figure S8. All recurrences avoided with adjuvant atezolizumab vs BSC where all events continue up to year 10, by province and recurrence type (10 years, 2024-2034)

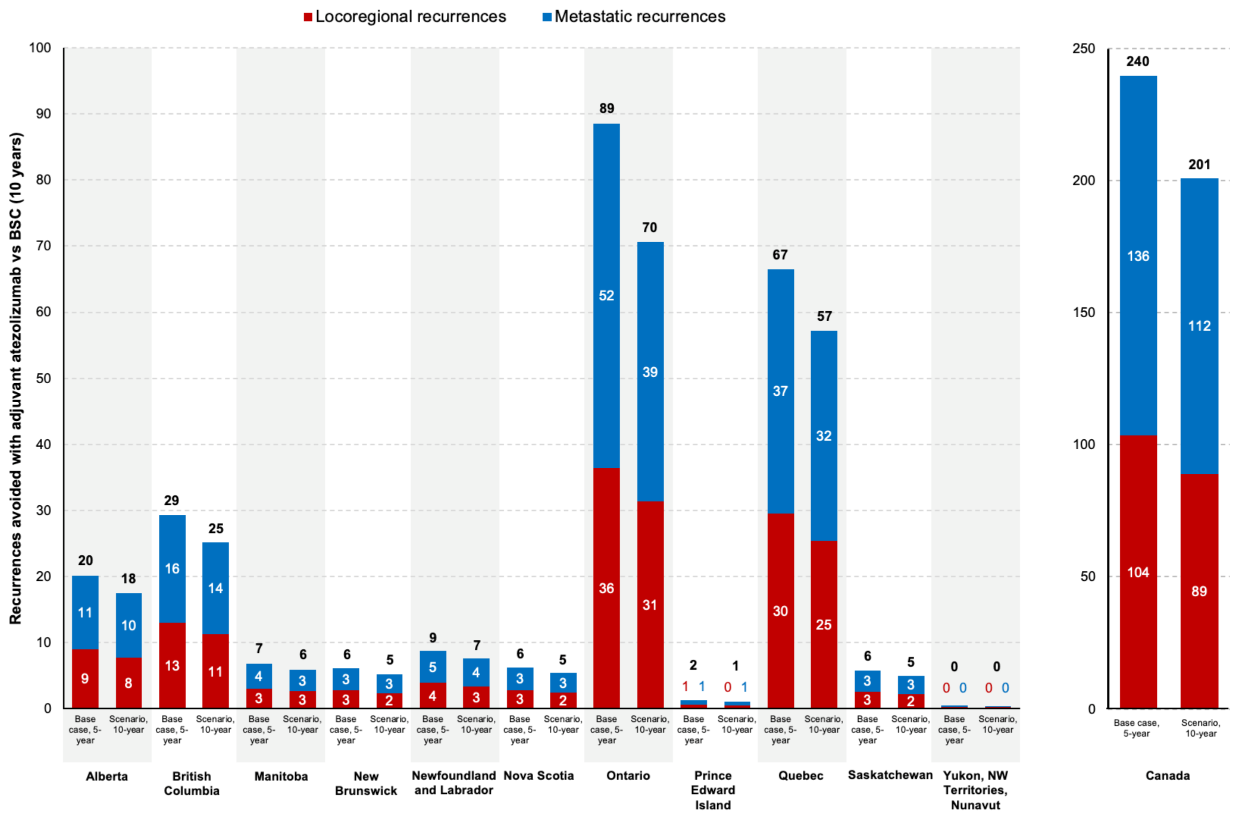

BSC, best supportive care.

Figure S9. Cost savings with adjuvant atezolizumab vs BSC where all events continue up to year 10, by province (10 years, 2024-2034)

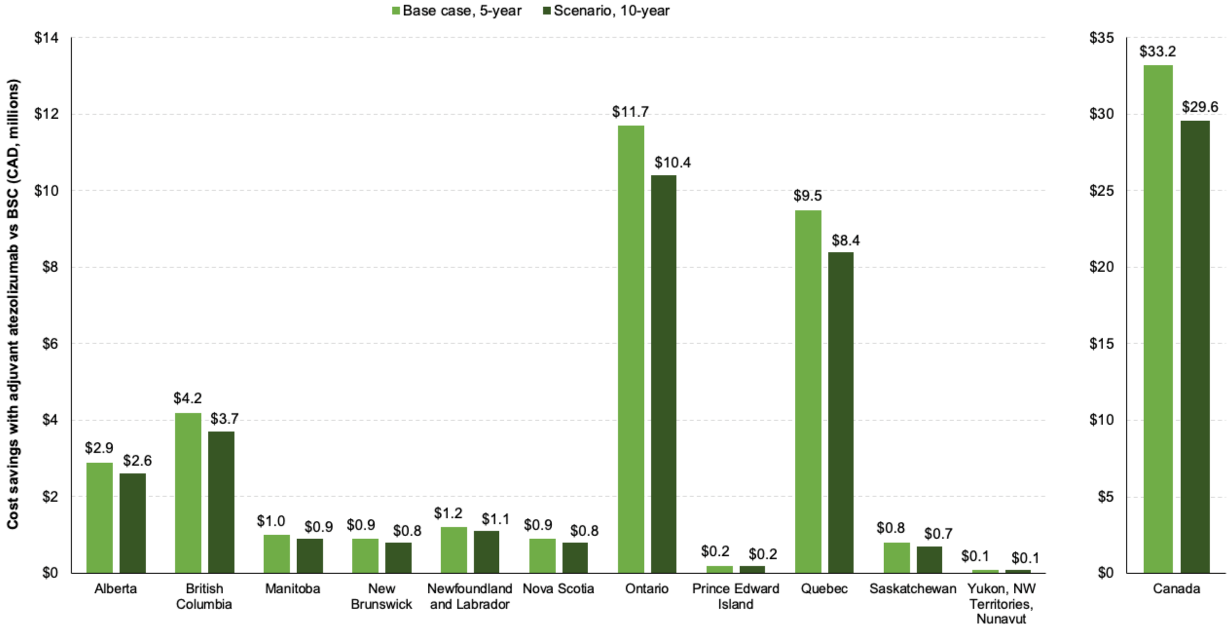

BSC, best supportive care.

Figure S10. All recurrences avoided with adjuvant atezolizumab vs BSC by DFS distribution and province (10 years, 2024-2034)

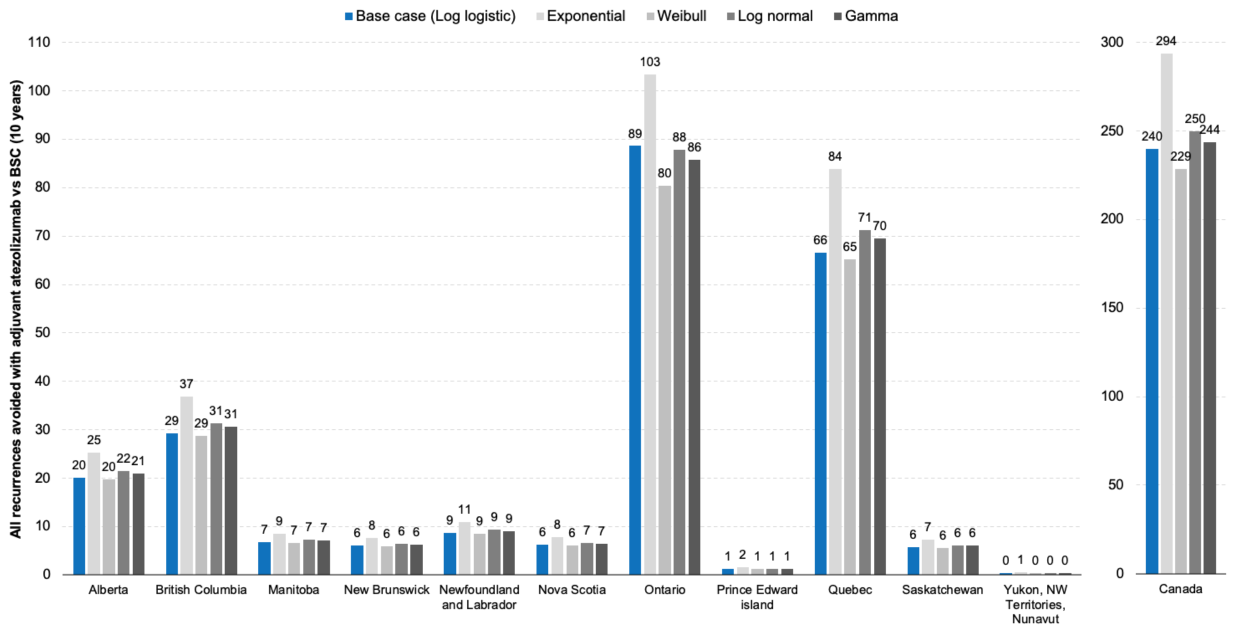

BSC, best supportive care; DFS, disease-free survival.

Figure S11. Cost savings with adjuvant atezolizumab vs BSC by DFS distribution and province (10 years, 2024-2034)

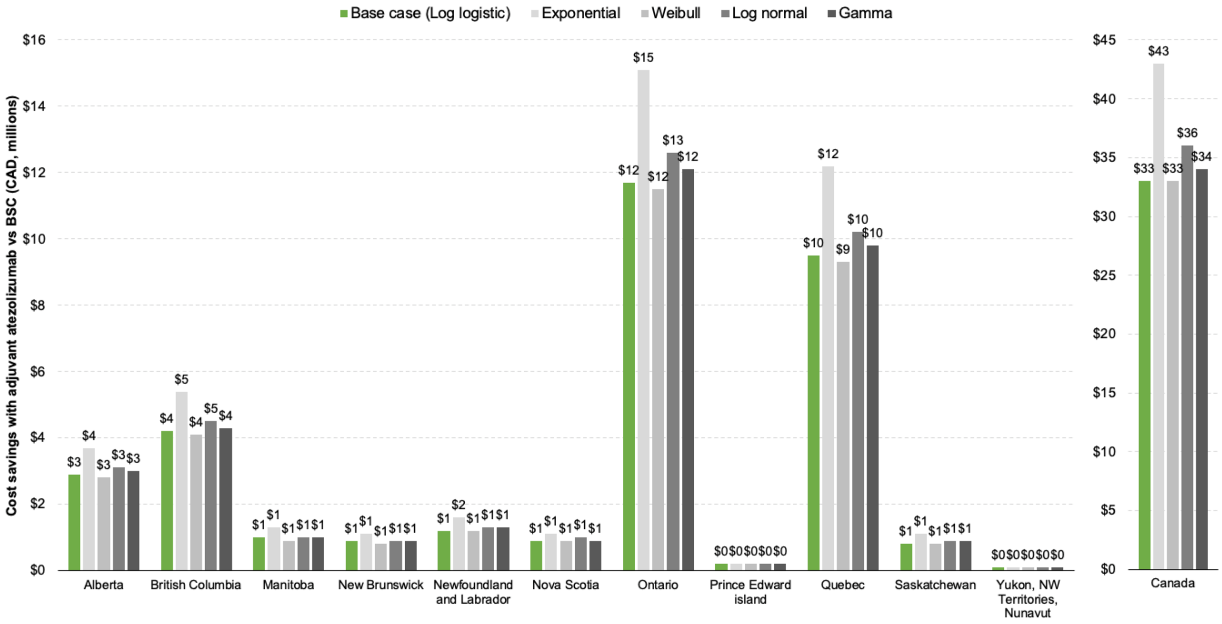

SUPPLEMENTARY MATERIAL REFERENCES

1. Felip E, Altorki N, Zhou C, et al. Adjuvant atezolizumab after adjuvant chemotherapy in resected stage IB-IIIA non-small-cell lung cancer (IMpower010): a randomised, multicentre, open-label, phase 3 trial. *Lancet*. 2021;398(10308):1344-1357. doi:10.1016/S0140-6736(21)02098-5
2. NICE. Durvalumab for Treating Locally Advanced Unresectable Non-Small-Cell Lung Cancer After Platinum-Based Chemoradiation [TA578]. Available online: https://www.nice.org.uk/guidance/ta578 (accessed on 14 April 2022).
3. NICE. Atezolizumab in Combination For Treating Metastatic Non-Squamous Non-Small-Cell Lung Cancer [TA584]. Available online: https://www.nice.org.uk/guidance/ta584 (accessed on 19 July 2023).
4. Ministry of Health. Schedule of Benefits. Physician Services under the Health Insurance Act. Available online: https://www.health.gov.on.ca/en/pro/programs/ohip/sob/physserv/sob_master.pdf (accessed on 20 July 2023).
5. CADTH. CADTH Reimbursement Review. Durvalumab (Imfinzi). Available online: https://www.cadth.ca/sites/default/files/DRR/2021/PC0234-combined-final.pdf (accessed on 20 July 2023).
6. IQVIA. IQVIA DeltaPA. Available online: https://www.iqvia.com/locations/canada/library/fact-sheets/iqvia-deltapa (accessed on 20 July 2023).
7. Antonia, S.J.; Villegas, A.; Daniel, D.; Vicente, D.; Murakami, S.; Hui, R.; Yokoi, T.; Chiappori, A.; Lee, K.H.; de Wit, M.; et al. Durvalumab after chemoradiotherapy in stage III non-small-cell lung cancer. N. Engl. J. Med. 2017, 377, 1919–1929. https://doi.org/10.1056/NEJMoa1709937.
8. Gandhi, L.; Rodriguez-Abreu, D.; Gadgeel, S.; Esteban, E.; Felip, E.; De Angelis, F.; Domine, M.; Clingan, P.; Hochmair, M.J.; Powell, S.F.; et al. Pembrolizumab plus chemotherapy in metastatic non-small-cell lung cancer. N. Engl. J. Med. 2018, 378, 2078–2092. https://doi.org/10.1056/NEJMoa1801005.
9. pCODR. Final Economic Guidance Report: Pembrolizumab (Keytruda) for Renal Cell Carcinoma. Available online: https://www.cadth.ca/sites/default/files/pcodr/Reviews2020/10185PembrolizumabRCC_fnEGR_NOREDACT-ABBREV_Post02Apr2020_final.pdf (accessed on 20 July 2023).

10. Scagliotti, G.V.; Parikh, P.; von Pawel, J.; Biesma, B.; Vansteensiste, J.; Manegold, C.; Serwatowski, P.; Gatzemeier, U.; Digumartia, R.; Zukin, M.; et al. Phase III study comparing cisplatin plus gemcitabine with cisplatin plus pemetrexed in chemotherapy-naive patients with advanced-stage non-small-cell lung cancer. J. Clin. Oncol. 2008, 26, 3543–3551. https://doi.org/10.1200/JCO.2007.15.0375.
11. Reck, M.; Kaiser, R.; Mellemgaard, A.; Doulliard, J.-V.; Orlov, S.; Krakowski, M.; von Pawel, J.; Gottfried, M.; Bondarenko, I.; Liao, M.; et al. Docetaxel plus nintedanib versus docetaxel plus placebo in patients with previously treated non-small-cell lung cancer (LUME-Lung 1): A phase 3, double-blind, randomised controlled trial. Lancet Oncol. 2014, 15, 143–155. https://doi.org/10.1016/S1470-2045(13)70586-2.
12. pCODR. Final Economic Guidance Report: Nivolumab (Opdivo) for Non-Small Cell Lung Cancer. Available online: https://www.cadth.ca/sites/default/files/pcodr/nivolumab_opdivo_nsclc_fn_egr.pdf (accessed on 20 July 2023).
